# Comparison of two percutaneous tracheostomy techniques, guide wire dilating forceps and Ciaglia Blue Rhino: a sequential cohort study

**DOI:** 10.1186/cc2907

**Published:** 2004-07-05

**Authors:** Bernard G Fikkers, Marieke Staatsen, Sabine GGF Lardenoije, Frank JA van den Hoogen, Johannes G van der Hoeven

**Affiliations:** 1 Department of Intensive Care, University Medical Centre Nijmegen, The Netherlands; 2 Department of Otorhinolaryngology, Head and Neck Surgery, University Medical Centre Nijmegen, The Netherlands

**Keywords:** intensive care unit, percutaneous tracheostomy, technique

## Abstract

**Introduction:**

To evaluate and compare the peri-operative and postoperative complications of the two most frequently used percutaneous tracheostomy techniques, namely guide wire dilating forceps (GWDF) and Ciaglia Blue Rhino (CBR).

**Methods:**

A sequential cohort study with comparison of short-term and long-term peri-operative and postoperative complications was performed in the intensive care unit of the University Medical Centre in Nijmegen, The Netherlands. In the period 1997–2000, 171 patients underwent a tracheostomy with the GWDF technique and, in the period 2000–2003, a further 171 patients with the CBR technique. All complications were prospectively registered on a standard form.

**Results:**

There was no significant difference in major complications, either peri-operative or postoperative. We found a significant difference in minor peri-operative complications (*P *< 0.01) and minor late complications (*P *< 0.05).

**Conclusion:**

Despite a difference in minor complications between GWDF and CBR, both techniques seem equally reliable.

## Introduction

Tracheostomy is usually performed in patients who need prolonged mechanical ventilation, frequent suctioning of bronchopulmonary toilet or have obstruction of the upper airway. The percutaneous tracheostomy is a minimally invasive, effective and reliable procedure and has become the alternative to surgical tracheostomy [1]. Almost all percutaneous procedures in The Netherlands are performed with one of the three following techniques: guide wire dilating forceps (GWDF) tracheostomy, Ciaglia Blue Rhino (CBR) tracheostomy, and sequential dilation tracheostomy (classic Ciaglia) [2]. We have extensive experience with the first two techniques [3,4]. This study is a sequel to our previous reports. Several studies have compared different percutaneous techniques [5-12], but because CBR is relatively new, a comparison with GWDF has been made only twice in two small prospective cohorts [5,12]. The strength of the present study is the large group of patients, so the incidence of relevant complications is more meaningful.

The aim of this study was to compare GWDF and CBR. The study not only focuses on the immediate peri-operative complications but also describes the long-term sequelae of both techniques.

## Methods

This is a retrospective analysis of all patients who underwent percutaneous tracheostomy in the University Medical Centre Nijmegen between March 1997 and April 2003. We compared the two historic data sets that we have published previously [3,4], but we specifically focused on the precise definition of early complications and long-term sequelae. Between March 1997 and February 2000 we performed percutaneous tracheostomy on 171 patients, using the GWDF technique. Between March 2000 and April 2003 we performed percutaneous tracheostomy on a further 171 patients, using the CBR technique. Indications, contra-indications and technique for percutaneous tracheostomy are standardised [3,4]. Patients or family gave informed consent before the procedure. Ethical approval from the institution's medical ethical committee was not obtained because the standard of care was provided and no other experimental treatments were introduced. Published data cannot be reduced to a single recognisable patient. All data were recorded prospectively on pre-designed forms. 'Procedure time' was defined as the time from incision to successful placement of the cannula. A 'peri-operative complication' was defined as a complication related to the procedure and occurring during or within 24 hours of the procedure. Postoperative complications were divided into 'complications while cannulated' and 'late complications'. A 'complication while cannulated' was defined as a complication occurring in the period between 24 hours after the procedure until removal of the cannula. A 'late complication' was defined as a complication occurring after removal of the cannula up to a follow-up of 3 years. Complications were divided into minor and major (see Tables 1, 2, 3). Moreover, complications were classified as procedure-specific and procedure-non-specific. Hypotension was defined as a systolic blood pressure of less than 90 mmHg. Hypoxaemia was defined as an arterial oxygen saturation of less than 90%. It was considered minor when lasting less than 5 min, and major when lasting 5 min or longer. Information regarding late complications was obtained by structured interviews with patients who were decannulated successfully. Patients or close relatives were asked about voice changes, dyspnoea, stridor, pain, and cosmetic problems. Patients were also asked to grade specific problems as absent, minor or major.

**Table 1:** Peri-operative complications

Complication	GWDF (*n *= 171)		CBR (*n *= 171)		*P*	Conversion to surgical tracheostomy	
				
	No.	%	No.	%		GWDF (*n *= 6)	CBR (*n *= 2)
No complications	128	74.9	100	58.5	<0.01		
Minor complications							
Procedure-specific							
Bleeding (local pressure)	11	6.4	24	14.0	0.04		
Difficult dilation	0		23	13.5	<0.01		
Difficult procedure	6	3.5	7	4.1	NS		
Subcutaneous emphysema	2	1.2	2	1.2	NS		
Cannula insertion difficult	0		3	1.8	NS		
Air leakage cuff	0		2	1.2	NS		
Procedure-non-specific							
Puncture endotracheal tube	9	5.3	8	4.7	NS		
Puncture posterior tracheal wall	4	2.3	2	1.2	NS		
Accidental detubation	1	0.6	3	1.8	NS		
Hypotension	1	0.6	2	1.2	NS		
Total	34	19.9	75	43.9	<0.01		
Major complications							
Procedure-specific							
Bleeding (exploration)	6	3.5	4	2.3	NS	2	
Bleeding (life-threatening)	1	0.6	1	0.6	NS		
Fausse route	2	1.2	1	0.6	NS		1
Oesophageal perforation	1	0.6	0		NS	1	
Cannula insertion impossible	3	1.8	0		NS	3	
Pneumothorax	0		3	1.8	NS		1
Total	13	7.6	9	5.3	NS		

^a^Some patients had more than one complication. CBR, Ciaglia Blue Rhino; GWDF, guide wire dilating forceps; NS, not significant.

**Table 2:** Complications while cannulated

Complication	GWDF		CBR		*P*
			
	No.	%	No.	%	
Surgical tracheostomy	6		2		
Lost to follow-up	1		0		
Available for analysis	164		169		
No complications	139	84.8	138	81.7	NS
Minor complications					
Bleeding (local pressure)	15	9.1	14	8.3	NS
Infection	4	2.4	6	3.6	NS
Granulation tissue around stoma	1	0.6	1	0.6	NS
Pain from stoma	1	0.6	0		NS
Tracheal oedema	0		1	0.6	NS
Subcutaneous emphysema	0		1	0.6	NS
Dyspnoea	0		1	0.6	NS
Total	21	12.8	24	14.2	NS
Major complications					
Bleeding (exploration)	0		2	1.2	NS
Bleeding (life-threatening)	0		0		NS
Stridor (with empty cuff)	2	1.2	0		NS
Cardiopulmonary resuscitation	1	0.6	0		NS
Cannula obstruction	1	0.6	3	1.8	NS
Hypoxaemia	0		2	1.2	NS
Total	4	2.4	7	4.1	NS

CBR, Ciaglia Blue Rhino; GWDF, guide wire dilating forceps; NS, not significant.

All data were analysed with Statistical Product and Service Solutions (SPSS) version 11.0. All variables were checked for normal distribution. Data are given as means ± SD or medians. Continuous variables were compared with Student's *t*-test or the Mann–Whitney test as appropriate. Bonferroni's correction for multiple comparisons was used. Categorisable variables were compared with the χ^2 ^test. A cut-off level of *P *< 0.05 was accepted as statistically significant.

**Table 3:** Late Complications

Complication	GWDF		CBR		P
			
	No.	%	No.	%	
Surgical tracheostomy	6		2		
Lost to follow up	5		6		
Still cannulated	0		3		
Deceased	53		60		
Available for analysis	107		100		
No complications	86	80.2	73	73.0	NS
Minor Complications					
Voice	9	8.5	22	22.0	<0.01
Cosmetic problems	10	9.4	2	2.0	0.04
Pain	0		2	2.0	NS
Total minor complications	19	17.9	26	26.0	NS
Major complications					
Stridor	2	1.9	1	1.0	NS

CBR, Ciaglia Blue Rhino; GWDF, guide wire dilating forceps; NS, not significant.

## Results

Demographic data are shown in Table 4. The procedure was successful in 165 of 171 patients (96.5%) in the GWDF group and in 169 of 171 patients (98.8%) in the CBR group. Most tracheostomies were performed by an intensivist or a fellow (under supervision). More procedures were performed by a fellow in the CBR group than in the GWDF group (51 versus 27, respectively; *P *< 0.01).

**Table 4:** Demographic data

Parameter	GWDF (*n *= 171)			CBR (*n *= 171)			*P*
			
	Mean	SD	Median	Mean	SD	Median	
Age (years)	57.5	18.2	62	57.5	18.4	62	NS
Male/Female	99/72			114/157			NS
Endotracheal intubation (days)	16.9	12.2	14	20.3	12.3	18	0.03
Procedure time (min)	9.1	8.3	5.0	10.8	10.5	7.0	NS
Cannulation time (days)	38.4	63.4	24	29.6	39.8	18	NS
Time in ICU (days)	39.4	29.8	33	44.1	38.3	34	NS

CBR, Ciaglia Blue Rhino; GWDF, guide wire dilating forceps; ICU, intensive care unit; NS, not significant.

### Peri-operative complications

Peri-operative complications are described in Table 1. In total, there were 47 peri-operative complications in 43 patients in the GWDF group, and 84 peri-operative complications in 71 patients in the CBR group (*P *< 0.05). This difference is explained by a greater number of difficult dilations (*P *< 0.01) and minor bleedings with the CBR technique. After the introduction of a Crile's forceps for blunt dissection of the pretracheal tissues preceding CBR, the procedure became much easier. In the GWDF group, 13 patients (7.6%) had a major complication, compared with 9 patients (5.3%) in the CBR group. All these major peri-operative complications were procedure-specific. One life-threatening bleeding in the GWDF group led to severe hypoxia at the end of the procedure. After removal of the cannula, large blood clots were suctioned from the trachea. There was no significant difference in the number of patients in whom conversion to a surgical tracheostomy was necessary. In the GWDF group, six patients underwent conversion to a surgical tracheostomy: one patient had a major venous bleeding after dilation of the trachea and the cannula could not be inserted. In another patient, arterial blood was aspirated and the procedure was terminated. In two patients, the trachea was difficult to locate, resulting in hypoxaemia and hypercapnia. In one patient the guide wire was placed correctly but the cannula perforated the posterior tracheal wall and entered the oesophagus. Surgical exploration confirmed rupture of the oesophagus, and the tracheo-oesophageal wall was immediately repaired. The post-operative course was uneventful. In the last patient the distance between skin and trachea was too large for the insertion of a cannula. In the CBR group two patients underwent surgical tracheostomy: in one patient the trachea was difficult to locate, and the cannula was placed pretracheally as a result of guide wire kinking. Another patient developed major bleeding and tension pneumothorax several hours after the procedure. After immediate drainage with a chest tube, surgical exploration showed that the tracheostomy tube had perforated the cricothyroid membrane. No deaths were seen after either procedure.

### Complications while cannulated

In total, 164 GWDF and 169 CBR patients were analysed for complications while cannulated (Table 2). Four major complications (2.4%) occurred in the GWDF group, and seven major complications (4.1%) in the CBR group. One patient in the GWDF group had an obstruction of the cannula by a mucous plug, leading to a cardiorespiratory arrest. Another patient sustained a cardiorespiratory arrest shortly after decannulation, possibly due to aspiration. Both patients were resuscitated successfully. Three patients in the CBR group had an obstruction of the cannula: one of them died on his first day on the ward, possibly owing to an obstructive blood clot in the cannula. The second patient had a mucous plug causing severe hypoxaemia. He received a minitracheotomy through the old tracheostomy opening. The third patient with an obstructed cannula was found in bed on the ward, having a respiratory arrest. The inner cannula, which was obstructed by a blood clot, was removed. The patient recovered uneventfully.

### Late complications

Of 164 patients in the GWDF group, 53 (32.3%) died with the cannula in place or within 1 week after decannulation, and five patients were lost to follow-up. One hundred and seven GWDF patients (62.6%) were decannulated successfully and analysed for late complications (Table 3). Of 169 CBR patients, 60 (35.5%) died with the cannula in place or within 1 week of decannulation, six patients were lost to follow-up, and three patients had the cannula still *in situ*. Finally, 100 CBR patients (58.5%) were analysed for late complications. There was no significant difference between both groups with regard to total late complications. All patients with voice problems were given the opportunity to consult an ENT specialist. None of these had an objective laryngeal abnormality explaining their voice problems. Patients with cosmetic problems relating to the tracheostomy scar were offered specialist consultation. Six GWDF patients underwent scar revision. Three patients developed a severe stridor after decannulation. In the GWDF group, an 83-year-old woman had tracheal stenosis and was treated with an endotracheal stent, and an 80-year-old woman was treated with laser for a granuloma just above the tracheostomy opening. In the CBR group, an 18-year-old man suffered from severe tracheal stenosis. He had a tracheal stent placed initially, but because of recurrence of the stenosis, a tracheal resection was necessary. The patient recovered uneventfully.

## Discussion

In this study we have compared two different techniques of percutaneous tracheostomy, GWDF and CBR. Both techniques are frequently used in The Netherlands and are replacing the surgical technique [2]. This study showed no significant differences in clinically relevant complications between the two techniques. This is in agreement with two other studies comparing these techniques [5,12]. Although the total number of complications in the two groups in the study of Ambesh and colleagues was not significantly different, the authors noticed an increased rate of minor peri-operative bleeding in the GDWF group [5]. This was balanced by an increase in the number of patients with one or more tracheal ring fractures in the CBR group (30%). The increase in major peri-operative bleeding with the GDWF technique might be explained by the poorly controllable dilation with the forceps [9]. Although the study of Añón and colleagues did not find any significant differences, in three of 26 patients in the GWDF group there was an inability to insert the cannula [12].

Several other studies comparing sequential dilation (classic Ciaglia) and CBR [6,8], and comparing sequential dilation and GWDF [7,9-11], have been described in the literature. Van Heurn and colleagues concluded that sequential dilation and GWDF are both reliable but that sequential dilation has fewer early complications than GWDF [7]. Nates and colleagues also preferred sequential dilation to the GWDF technique, because of fewer surgical complications, less peri-operative and postoperative bleeding, and easier use [9]. Añón and colleagues found a comparable complication rate, but the procedural time of the GWDF method was significantly shorter [10]. Unfortunately, comparing these studies is difficult because complications were not defined uniformly.

In our study, a major complication while cannulated was obstruction of the cannula, which occurred in four patients. These figures correspond to the prevalence of cannula obstruction in the literature (0.3–3.5%) [13-15]. Strict adherence to nursing protocols and a low threshold for cleaning the inner cannula should be the standard of care in the intensive care unit. An outreach team from the intensive care unit should visit patients, discharged to the general ward with a cannula in place, on a daily basis.

There are only few data available concerning late complications of percutaneous tracheostomy. Unfortunately, many confounders might be present, such as the disease process itself, the duration of endotracheal intubation, and other treatments in the intensive care unit (such as sedation or physical therapy). Moreover, both patients and caregivers often interpret late complications subjectively. The total number of late complications in our study was not significantly different between the two groups. Subjective voice changes and hoarseness were more frequent in the CBR group (*P *< 0.01). An explanation might be the longer mean endotracheal intubation time, because this is possibly the most important cause of voice problems. With sequential dilation tracheostomy, the incidence of voice problems ranges between 0% and 21% [16-22]. More patients in the GWDF group complained of cosmetic problems. Only a few studies have mentioned cosmetic complaints, but differences of opinion between patient and caregiver are frequent [23]. In each group in our study, one patient developed a critical symptomatic tracheal stenosis. More patients might have had an asymptomatic tracheal stenosis, but because no additional diagnostic tests such as computed tomography or magnetic resonance imaging scans were performed, the actual incidence is unknown. Several studies have incriminated the GWDF technique as a cause of tracheal stenosis, but no studies with the CBR have been described. The incidence varied from 0% to 63% [18,23-27]. Most of these tracheal stenoses were asymptomatic.

Several factors might decrease the strength of our conclusions. First, the study used historical data sets with a sequential design; a time bias is therefore possible. As experience with percutaneous tracheostomy increases, the number of complications will decrease, even if another technique is used, although in our study this might well have been balanced by the fact that over time more fellows performed the procedure. Second, scoring of the peri-operative complications by different physicians might be variable because of different interpretations. Despite these shortcomings, we conclude from our study that, although the CBR technique has more minor peri-operative complications, the two techniques are comparable. More prospective, randomised studies are required to compare these different tracheostomy techniques adequately. We are currently conducting a prospective, randomised study in which we compare GWDF and CBR tracheostomies; we are specifically looking for the occurrence of precisely defined early and late complications. The occurrence of tracheal stenosis will be analysed using the forced oscillation technique and magnetic resonance imaging.

## Key messages

• GWDF and CBR tracheostomy seem equally reliable.

• Major peri-operative complications occur in 5.3–7.6% of patients.

• Late complications are rare

## Competing interests

None declared.

## Abbreviations

CBR = Ciaglia Blue Rhino; GWDF = guide wire dilating forceps.
